# Cost-effectiveness of Neonatal Hearing Screening Programs: Systematic Review

**DOI:** 10.1055/s-0043-1776703

**Published:** 2024-04-09

**Authors:** Luíza Silva Vernier, Carolina Pereira Fernandes, Pedro Pablo Skorin, Audrei Thayse Viegel de Ávila, Daniela Centenaro Levandowski

**Affiliations:** 1 Department of Postgraduate Program in Health Sciences, Universidade Federal de Ciências da Saúde de Porto Alegre, Porto Alegre, RS, Brazil; 2 Department of Speech Therapy, Universidade Federal de Ciências da Saúde de Porto Alegre, Porto Alegre, RS, Brazil; 3 Department of Economics and International Relations, Universidade Federal do Rio Grande do Sul, Porto Alegre, RS, Brazil; 4 Department of Speech Therapy, Hospital de Clínicas de Porto Alegre, Porto Alegre, RS, Brazil; 5 Department of Psychology, Universidade Federal de Ciências da Saúde de Porto Alegre, Porto Alegre, RS, Brazil

**Keywords:** cost-effectiveness, hearing, neonatal screening, systematic review

## Abstract

**Introduction** Universal newborn hearing screening (UNHS) has been widely and strongly advocated as an early detection strategy for hearing loss in children. This intervention aims to prevent delays in speech and language development, which, in turn, has long-term beneficial effects on the social and emotional development and quality of life of individuals. However, the implementation of UNHS programs is circumstantial in different settings, for different reasons.

**Objectives** The present systematic review aimed to identify whether the implementation of UNHS programs are cost-effective, as well as their variations by localities.

**Data Synthesis** A search was conducted in seven databases: PubMed (Medline), Scopus, Web of Science, Embase, CINAHL, Lilacs, and Cochrane Library. Studies that included a cost analysis of UNHS programs were eligible for inclusion. Studies on evaluations of preschool or school-based programs only were excluded, among others. A total of 1,291 records were found. Of these, 23 articles were analyzed in full. All articles identified the cost-effectiveness of the UNHS programs implemented. Regarding the UNHS protocols, a wide variation was observed in all aspects: tests used, period established between tests and retests, professionals responsible for screening, environment, and criteria for defining hearing loss, limiting the generalization of this information. All studies presented values related to the expenses with the program, but none of them showed statistical elements for the described analyzes or any theoretical basis for such.

**Conclusion** It is necessary to estimate local specific issues, as well as the accuracy of the chosen tests and the NHS protocols used, so that more accurate analyzes on cost-effectiveness are possible.

## Introduction

Hearing loss is the fourth largest factor for years lived with physical disability in a worldwide analysis. 1 The World Health Organization (WHO) estimates that, in 2050, ∼ 466 million people worldwide will have a disabling hearing loss (6.1% of the world population) and that almost 34 million (7%) will be children. 2 The prevalence of hearing impairment in neonates is ∼ 2 in every 1,000 live births; in ∼ 2/3 of these, the alterations are bilateral. 3

The impacts of hearing loss may extend throughout life. In child development, for example, it is possible to observe delays in language and/or speech, changes in school performance, personal-social maladjustments, and emotional disorders. 4 In adolescence and adulthood, there are limitations in social relationships, employment opportunities, and an early onset of cognitive decline.

In addition to the clinical effects of untreated hearing loss, its economic cost is substantial. The estimated annual global costs of untreated hearing loss in the healthcare sector alone exceed US$ 100 billion. When productivity loss is included, this cost increases to US$ 750-790 billion annually. 5 As such, prevention appears to be the most cost-effective way to lessen the high and growing impact of hearing loss. 1

Given this, we realize the importance of early identification and intervention in hearing impairment in infants. To this end, Universal Newborn Hearing Screening (UNHS) programs have been widely implemented. 6 The goal of these programs is to detect and rehabilitate all infants with hearing loss early, keeping false-positive result rates low to avoid unnecessary costs and decrease parental concern. The collection of data by these UNHS programs can support managerial decision making, as it allows monitoring and evaluation of the performance of the evaluated infants and programs. 3 Although these programs are established and standardized in most developed countries, expansion efforts to implement UNHS in other countries continue to exist.

To assess the benefits of implementing strategies or programs, a cost-effectiveness analysis should be conducted, allowing decision-makers to clearly understand the trade-offs in costs, harms, and benefits between alternatives, which should be combined into a single metric, the Incremental Cost-Effectiveness Ratio (ICER). This metric can also be used to inform decision-making when there are limited resources. Therefore, we understand that one of the relevant factors to determine the success of a UNHS program is the cost-effectiveness ratio 7 to identify the factors that affect its performance as a whole. 8

Public policy makers have a position regarding the attention given to cost-effective interventions in hearing health, aiming to reduce the consequences of hearing loss. 9 The analysis of these data can gather information for resource allocation and potentiate investment and prioritize interventions. 1 But many countries still do not include UNHS programs in their health agenda, partly because they are considered too expensive or because their value is questioned. 10 In countries where the implementation of UNHS programs takes place, there are variations in the approach and methods used, which can be attributed to a difference in available resources, financial or technological, but also to the lack of universal guidelines to be followed, to ensure a consistent approach in the implementation of these programs. 11

Therefore, the present systematic review aimed to identify, in the literature, whether the implementation of UNHS programs is cost-effective, as well as their variations by location. We also tried to identify the differences between the protocols used, as well as the quality indicators of UNHS programs.

## Methods

### Protocol

The present review was registered in the International Prospective Register of Systematic Reviews (PROSPERO), under the number CRD42021257857, and is presented according to the PRISMA-ScR (Preferred Reporting Items for Systematic Reviews and Meta-analyzes - Extension for Scoping Reviews) guidelines. 12 13

A protocol for the literature search was structured ( Supplementary Material Appendix 1 ).

### Eligibility Criteria

The design of the studies had no restrictions. On the other hand, to be included in the present review, studies had to be original and present a formal economic analysis of UNHS programs, with descriptions of costs, cost analyzes, descriptions of cost outcomes or complete economic evaluations. Studies with evaluations of Hearing Screening programs for children in general, and not right after birth; that aimed to compare two or more protocols to perform UNHS; that presented the clipping of only one stage of UNHS; searches made based on simulated populations, and articles that limited the population to perform Neonatal Hearing Screening (NHS) were excluded.

### Sources of Information

**Fig. 1:**
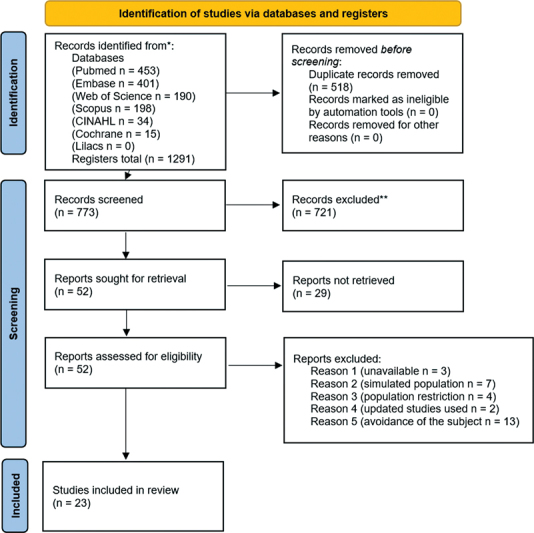
PRISMA 2020 flow diagram for new systematic reviews that included searches of databases and registers only.

Seven scientific databases were considered for the searches: PubMed (Medline), Scopus, Web of Science, Embase, CINAHL, Lilacs, and Cochrane Library. These databases were independently and simultaneously consulted by two researchers in May 2021. There was no limitation regarding publication date, language and/or geographical location. All records were considered as eligible for inclusion if they had an abstract. After reading the titles and abstracts, the included articles were forwarded for analysis of the full text. From the exclusions made at this stage, the reference lists of all eligible studies were examined to include any additional studies relevant to the objectives of the present study, provided they met the eligibility criteria. ( Fig. 1 )

### Search Strategy

The descriptors were chosen through Medical Subject Heading (MeSH) and were integrated into the search strategy, being related to the theme of the study: Newborn Screening, Hearing and Cost-Effectiveness. The full search algorithms for each database can be found in Supplementary Material Appendix 2 . The overall search strategy was devised by the study authors, consisting of: ( *Neonat* * *screening* * OR *Newborn* * *screening* *) AND ( *Hear* *) AND ( *Costs and Cost Analysis* OR *Cost Control* * OR *Economics* OR *Cost-Benefit Analyses* * OR *Cost-Effectiveness Evaluation* ).

### Study Selection Process

Two authors of the study simultaneously, independently, and blindly performed the initial searches in the databases. After the elimination of duplicate records, all titles and abstracts of the articles were independently evaluated to verify the possibility of inclusion, according to the eligibility criteria. Differences regarding inclusion or exclusion were resolved with the evaluation of a third author, who acted as a judge. The same happened in the phase of inclusion/exclusion of articles after full reading.

### Data Mapping Process

The data from the selected articles were extracted for registration and compilation of information in a spreadsheet made for the present study in Microsoft Excel (Microsoft Corporation, Redmond, WA, USA).

### Evaluation of Data and Results

All eligible articles evaluated UNHS programs and contained data such as the region of implementation of the program analyzed, components of the screening protocol followed by professionals, the economic analyzes performed and the cost-effectiveness perspective of the program. The primary outcome of interest was whether the implemented UNHS programs were evaluated as cost-effective, supported by economic cost-effectiveness relationships. Monetary value rates were reported when available. Items without dollar costs had the values converted to this currency using the Purchasing Power Parity (PPP) rate. 14 After the conversation to dollar, the implicit price deflator of gross domestic product 15 was used to adapt prices to 2012 levels, because the last economic analysis of the selected articles is from that year, minimizing distortions in the evaluation.

### Results Overview Method

No quantitative analyzes and meta-analysis were performed in the present review due to the heterogeneity of the eligible studies and the scarce data available on cost-effectiveness. A qualitative analysis of the findings of the reviewed studies was performed individually, comparing the outcomes of interest to the present study.

### Quality of Evidence

The Consolidated Health Economic Evaluation Reporting Standards (CHEERS) 16 parameters were used to assess the methodological quality of the reviewed studies. Each cost-effectiveness analysis reported by each article included in the review was evaluated considering the CHEERS checklist, a tool developed by the International Society for Pharmacoeconomics and Outcomes Research, with 24 items subdivided into 6 main categories: title and abstract; introduction; methods; results; discussion; and other. The items analyzed in each reviewed study were binarily scored. Each study received an aggregate score according to adherence to the criteria and any methodological concerns regarding each study were described.

## Results

### Evidence Selection

A total of 1,291 records were found. Initially, the duplicate records were removed, leaving 773 articles for analysis of the title and abstract. After the initial reading, 721 articles were excluded for evading the proposed theme ( *n* = 518), being reviews ( *n* = 121), being in formats other than articles ( *n* = 69) or unavailable ( *n* = 13), resulting in 52 studies for the full text analysis. After their full-text reading, 29 articles were excluded for not meeting the eligibility criteria for cost-effectiveness analysis of UNHS programs. Therefore, 23 articles were included in the present review.

### Population

As one of the inclusion criteria adopted, all studies performed a cost-effectiveness or economic analysis of some UNHS program. The studies included in the present review were published between 1995 and 2017 and depicted data from several countries, especially United States of America (9 studies). But the included studies also covered countries from other continents, being characterized by heterogeneity. ( Table 1 ).

**Table 1:** Characterization of the selected articles according to authorship, year, country of publication, place and period of analysis, and study design

Author	Year	Place of analysis of the NHS data	Period of analysis of NHS data	Delineation
Chen et al. 17	2017	Maternity hospitals in Shanghai, China	1 ^st^ March/2002 - 31 ^st^ December/2012	Retrospective
Wong et al. 18	2017	Region of Jinotega, Nicaragua (eight municipalities)	July/2004 - December/2004	Retrospective
Gupta et al. 19	2015	Neonatal tertiary care unit of All India Institute of Medical Sciences, Delhi, India	May/2011 - June/2012	Prospective
Bevilacqua et al. 20	2010	Hospital Público de Bauru, São Paulo, Brazil	Unspecified (duration of 3 years)	Retrospective
Mezzano et al. 21	2009	Region Liguria, Italy (13 birthing centers)	February/2002 - December/2004	Retrospective
Olusanya *et al.* 22	2009	Hospital and a community in central Lagos, Nigeria	May/2005 - April/2006 (40 weeks)	Retrospective
Ciorba et al. 23	2008	University Hospital of Ferrara, Italy	January/2000 - December/2006	Prospective
Cao-Nguyen et al. 24	2007	Geneva University Hospital, Switzerland	1995 February/2000 - 2004	Retrospective
Lin et al. 25	2005	Mackay Memorial Hospital, Taiwan	November/1998 - December/2004	Retrospective
Connolly et al. 26	2005	Jackson University Medical Center, Mississippi, USA	January/1997 - January/2002	Retrospective
Messner et al. 27	2001	Lucile Packard Children's Hospital of Stanford, Palo Alto, USA	1 ^st^ April/1998 - 31 ^st^ August/1999	Prospective
Gorga et al. 28	2001	Local hospital (not specified), Omaha, Nebraska, USA	1 ^st^ June/1998 - 31 ^st^ May/2000	Retrospective
Isaacson 29	2000	Department of Audiology, Temple University Hospital and Temple University Children's Medical Center, Philadelphia, Pennsylvania, USA	15 January/1998 - 1 February/1999	Retrospective
Kanne et al. 30	1999	Madigan Army Medical Center, Tacoma, Washington, USA	1 ^st^ April/1995 - 30 ^th^ June/1996	Retrospective
Bantock et al. 31	1998	Local Health Centre (not specified), London, UK	June/1995 - May/1996	Prospective
Mason *et al.* 32	1998	Kaiser Permanent Medical Center, Hawaii, USA	March/1992 - February/1997	Retrospective
Weirather et al. 33	1997	Logan Regional Hospital, Utah, USA	February/1996 - March/1996	Prospective
Barsky-Firkser et al. 34	1997	Saint Barnabas Medical Center, New Jersey, USA	1 ^st^ January/1993 - 31 ^st^ December/1995	Retrospective
Watkin 35	1996	Whipps Cross Hospital, London, UK	1992–1995	Retrospective
Maxon et al. 36	1995	Women and Children's Hospital, Providence, Rhode Island, USA	1 ^st^ July/1993 - 31 ^st^ December/1993	Retrospective
Abdul et al. 37	2012	Hamad Hospital and maternity ward of Al Khor General Hospital, Doha, Qatar	2005–2010	Retrospective
Khandekar et al. 38	2006	Different regions of Oman (not specified), Oman	January/2002 - December/2003	Retrospective
González De Aledo Linos et al. 39	2005	Public Hospitals (2) and Private Hospitals (1) in Cantabria (not specified), Cantabria, Spain	April/2001 - March/2003	Retrospective

### UNHS Protocols

Variations and divergences were recorded between the protocols used to perform UNHS in the different sites and variations in their structures. Information on the team responsible for performing the UNHS was not present in four studies; in the others, this data was present, and the types of professionals varied, for example otolaryngologist, audiologist, and nurse.

In each study, the rates of coverage, referrals for retesting and diagnosis, identified hearing loss, and false-positive results were investigated. All reported program coverage (rates that were reported ranged from 25.68 to 99.5%); on the other hand, the least presented information was the false-positive rate.

The data on the UNHS flow varied, but most studies pointed out the execution of the first test within the recommended period, that is, before hospital discharge. Screening procedures also varied regarding the tests used, screening algorithms, audiometric frequencies tested, expected time between test and retest, hearing loss definition criteria, and frequencies indicated for screening. The Otoacoustic Emissions (OAE) was the most used screening test, followed by the Automatic Brainstem Auditory Evoked Potential (BAEP-A). All these data are presented in detail in Table 2 .

**Table 2:** Information on protocols and coverage of the Newborn Hearing Screening Programs analyzed in the selected studies

Author	Protocol used	Protocol description	Professional /team responsible	Coverage	% retest	% diagnosis	% hearing loss	% false positive
Chen et al. 17	1 ^st^ and 2 ^nd^ phase: OAE 3 ^rd^ phase: BAEP-A	Phase 1: initial screening in hospital before discharge with OAE (within 3 days of birth). Phase 2: new screening (retest) with OAE of infants with possible hearing loss at 42 days of age. Phase 3: performance of a diagnostic test with BAEP-A from 3 to 6 months of age for those who failed retesting. Phase 4: medical intervention (compensation or reconstruction of hearing by fitting hearing aids or cochlear implants). Phase 5: language rehabilitation training in the child's first 6 years. Phase 6: Inclusive school education.	Audiologist, speech therapist, and nurse.	93.60%	10.58% (178.005)	1.01% (17.061)	0.15% (2.616)	6.25% (105.205)
Wong et al. 18	1 ^st^ and 2 ^nd^ phase: DPOAE 3 ^rd^ phase: BAEP	If the baby failed the initial screening (done with DPOAE), the mother was given a follow-up date at the clinic in Jinotega to repeat the screening with DPOAE and to have tympanometry done. Infants who failed the retest were examined by the otolaryngologist and referred for a BAEP.	N/A.	640 screened	5.94%	0.31%	N/A	N/A
Gupta et al. 19	1 ^st^ and 2 ^nd^ phase: BAEP-A 3 ^rd^ phase: diagnostic BAEP	The 1 ^st^ screening was performed when the infant was stable, preferably between 24-48 hours after birth. Infants who failed the 1 ^st^ screening (unilateral or bilateral) were screened again within 1 week. The 2 ^nd^ screening was performed in BE, even if the initial screening had “failed” in only 1 of the ears. Infants who failed the 2 ^nd^ screening (unilateral or bilateral) were referred for conventional BAEP at the outpatient clinic.	ENT technician, audiologist	84%	12.05%	2.10%	0.22%	N/A
Bevilacqua et al. 20	1 ^st^ and 2 ^nd^ phase: TOAE	The initial screening was performed 24 hours after birth. Babies who failed the 1 ^st^ phase performed the outpatient screening, as well as those who were not screened before discharge, in no more than 20 days after birth. The “pass” result is only considered when the presence of TOAE in the 2 screening phases is observed, in BE. In case of a “fail” result, the baby was submitted to diagnostic follow-up and intervention tests at the Audiology and Speech Therapy Outpatient Clinic at SPU, consisting of an otorhinolaryngological evaluation, child and family history, behavioral, electroacoustic and electrophysiological tests (BAEP and Stable State). Infants were discharged from the program if their hearing was normal and there was no evidence of risk factors in the child's history. On the other hand, infants with a history of risk factors were submitted to a reevaluation at, at most, 12 months.	Audiologist and otolaryngologist	90.52%	22.20%	3.33%	14.70%	14.48%
Mezzano et al. 21	Babies without RFHL: 1 ^st^ phase: OAE 2 ^nd^ phase: BAEP Infants with RFHL: BAEP	All babies without risk were submitted to a 1 ^st^ OAE test 48 to 72 hours after birth. NBs who failed the 1 ^st^ test were submitted to a 2 ^nd^ test in the 3 ^rd^ week of life, at the same birth center. The next level screening involved the assessment with BAEP of the selected population (babies without risk who failed 2 OAE) in the 3 ^rd^ month of life.	N/A.	32,502 screened	9.50%	1.05%	4.20%	N/A
Olusanya *et al.* 22	1 ^st^ phase: TOAE 2 ^nd^ phase: BAEP	Initial screening with TOAE, followed by screening in the 2 ^nd^ phase with BAEP for all NBs who failed the 1 ^st^ test before hospital discharge. The performance of BAEP in the program held in the community was scheduled 1 week after the TOAE failure. All infants who failed BAEP (in both programs) were referred for a diagnostic evaluation within 1 month, which consisted of tympanometry, BAEP and/or free-field visual reinforcement audiometry (infants > 6 months old).	Nursing assistant and support staff.	3,333 screened (1,330 in hospital and 2,003 in the community)	Hospital: 32.20%. Community: 14.30%.	Hospital: 3.30%. Community: 4.10%.	52 (7 in hospital and 45 in the community)	N/A
Ciorba et al. 23	Regular nursery: 1 ^st^ and 2 ^nd^ phase: OAE 3 ^rd^ phase: BAEP-A Neonatal ICU Nursery: 1 ^st^ and 2 ^nd^ phase: OAE 3 ^rd^ phase: BAEP-A	- Neonatal ICU nursery: Screening with OAE was performed after the 32 ^nd^ week of age. In case of failure, the baby was referred for retesting. If the result of the OAE retest was a failure, BAEP-A was performed after 1 day. In case of failure of BAEP-A, a complete audiological evaluation with a clinical BAEP was scheduled and an electrocochleography test was usually suggested. - Regular nursery: Testing typically occurred 36 to 48 hours after birth. An acceptable OAE response in BE was required to pass. In case of failure, referral for retesting occurred. In case of retest failure, a clinical assessment with BAEP was scheduled up to 30 days later.	N/A.	Regular nursery: > 90% Neonatal ICU nursery: 1,016 screened	Regular nursery: 12.20%. Neonatal ICU Nursery: 14.70%	Regular nursery: 0.78%. Neonatal ICU nursery: 10.50%.	Regular nursery: 0.19%. Neonatal ICU nursery: 2.20%.	Regular nursery: 11.40%. Neonatal ICU nursery: 4.10%.
Cao-Nguyen et al. 24	2000-2002: 1 ^st^ phase: TOAE 2 ^nd^ phase: TOAE 3 ^rd^ phase: DPOAE and BAEP 2002-2004: 1 ^st^ phase: BAEP	It was considered successful when the NB “passed” in 1 ear. If the baby failed the OAE, the test was repeated, if possible, before discharge from the maternity or a few days or weeks later at the ENT department. If they failed the 2 ^nd^ test as well, the auditory evaluation was completed with DPOAE and BAEP. Medium latency tests and behavioral evaluations were performed for diagnosis. Hearing evaluations were repeated when parents or pediatricians suspected a hearing alteration, and again at school, at 4 years of age.	Nurses and otolaryngologist	96.70% (in 2000) and 99.50% (following years)	7.51%	1.51%	1.71%	N/A
Lin et al. 25	11/1998-01/2004 (“initial stage”): TOAE + DPOAE 01/2004 - 12/2004 (“final stage”): TOAE + BAEP + DPOAE	Initial stage: Screening performed 48 hours after birth. Babies who did not pass the initial screening had the chance to repeat the TOAE screening two more times before being discharged from the hospital. If the failure persisted, the newborns underwent DPOAE testing and tympanometry. If they failed, they underwent diagnostic evaluation with BAEP, Stable-State ABP, behavioral observations test (with noise makers, gurgling tones, narrow band noise and live voice) or VRA. Final stage: Infants who did not pass the initial screening with TOAE had BAEP before discharge from the hospital. In case of failure, they underwent DPOAE and tympanometry. If they failed again, a diagnostic evaluation was performed with the same tests of the previous stage.	N/A.	21,273 screened (18,260 in the early stage and 3,013 in the late stage).	N/A	Initial stage: 5.80%. Final stage: 1,80%	Initial stage: 0.45%. Final stage: 0,33%	N/A
Connolly et al. 26	BAEP	A test was performed right after birth and another one before discharge. Neonates who failed the 2 ^nd^ screening were referred for retesting as outpatients or for audiological and medical evaluation. All high-risk patients were referred for audiological follow-up, regardless of the initial results. Babies who failed a 2 ^nd^ BAEP (with extended hospital courses) were retested randomly with the BAEP, at the nursing team's discretion, until they passed or until hospital discharge. There was neither several tests determined by the hospital, nor a minimum time established between retests (variation between 1 day and 1 month). Therefore, the number of screenings before discharge depended on the nursing team. Retesting could be in BE or in only one ear.	Nurses.	> 99%	N/A	3.00%.	0.44%	3.90%
Messner et al. 27	1 ^st^ and 2 ^nd^ phase: BAEP-A 3 ^rd^ and 4 ^th^ phase: TOAE 5 ^th^ phase: diagnostic BAEP	In regular nurseries, BAEP-A screening was usually performed within 24 hours after birth and could occur between 48 and 72 hours of life. A neonate needed to pass the screening in BE for the parents to receive a green card for release to go home. If the NB failed one or BE, the parents received a pink “referral” card. If the NB was discharged without having done the screening, a yellow card, “not screened”, was given to the family, who was contacted to return for a screening. Screening in the NICU and ICU was conducted with the BAEP-A and occurred before discharge, and the neonate should be at least 34 weeks gestation and stable to be tested. In case of failure, the NB would repeat the test with the BAEP-A. If it failed again, it was referred for screening with TOAE. The NB who failed this test would be referred to the hospital pediatric audiology clinic for a new TOAE test. If the baby failed all screenings, a diagnostic evaluation, including tympanometry and diagnostic BAEP, was performed.	Volunteers, nurses, and pediatric audiologists.	Regular nursery: 91%. ICU + ICU: N/A	Regular nursery: 11%.	N/A	Total: 21	N/A
Gorga et al. 28	1 ^st^ phase: DPOAE 2 ^nd^ phase: DPOAE 3 ^rd^ phase: BAEP	All newborns underwent an examination with DPOAE. Those who did not pass in BE performed another examination with DPOAE. In case of persistent failure, the newborns were referred for BAEP. All tests were performed before hospital discharge. With some exceptions, all babies in the regular nurseries were tested on the 1 ^st^ or 2 ^nd^ day of life. Babies in the Neonatal ICU were tested closer to discharge. Babies who failed BAEP were referred as outpatients for further tests.	Audiologist.	97.50%	7%	1.7%	0.08%	N/A
Isaacson 29	1 ^st^ and 2 ^nd^ phase: TOAE 2 ^nd^ phase: BAEP	Babies in the regular nursery were tested from 16 hours after birth. Infants in the Neonatal ICU were screened in the last days before discharge. Babies who failed the screening and had no risk factors were scheduled for retesting with TOAE and auditory response threshold test with BAEP, between 4 and 6 weeks after discharge. Babies who passed the screening but had risk factors were instructed to schedule appointments for repeat TOAE testing, behavioral audiometry, and tympanometry for infants aged 6 to 9 months. Babies who failed screening and had risk factors were scheduled for retesting with TOAE and BAEP, 4 to 6 weeks after discharge.	Audiologist, secretary, and pediatric otolaryngologist.	95%	N/A	8.20%	0.68%	N/A
Kanne et al. 30	1 ^st^ and 2 ^nd^ phase: TOAE 3 ^rd^ phase: BAEP	Babies admitted to the Neonatal ICU were screened between 24 and 72 hours before hospital discharge. Babies in the regular nursery were screened at the first outpatient visit, at 2 weeks of age. Infants who failed were referred for retesting. Those NB who failed the first 2 phases were referred for diagnostic evaluation with BAEP. In case of new failure, the patients were referred for medical evaluation.	Audiologist, management team.	90.20%	7.82%	2.05%	0.20%	8.70%
Bantock et al. 31	1 ^st^ and 2 ^nd^ phase: TOAE 3 ^rd^ phase: BAEP	At 3 to 4 weeks of age, babies were to go to a specialized clinic to be screened with TOAE. Those who failed in 1 or BE were to repeat the test 1 or 2 weeks later. Those in whom OAE had not yet registered in BE were referred to the 2nd level clinic for BAEP testing. The interval between the 2nd OAE failure and the BAEP test was usually 1-2 weeks. Infants who did not obtain responses at 80 dBnHL levels were immediately referred to a tertiary ENT/audiology center. Management of cases with responses present between 50-70 dBHL was decided individually. NBs who had absent TOAEs in 1 ear or emissions at only a few frequencies in BE on the 2 ^nd^ TOAE test were followed up at the 2 ^nd^ level clinic. At 4 to 7 months of age, tympanometry could be used to identify infants who had persistent otitis media with effusion.	Nurse, and administrative assistant.	69.97%	18.40%	0.41%	0	N/A
Mason *et al.* 32	1 ^st^ phase: BAEP-A 2 ^nd^ phase: Diagnostic BAEP	Healthy babies, hospitalized for 24 to 36 hours, were tested between 3 and 36 hours of life. Babies in the neonatal ICU were tested before discharge (between 2 and 90 days or more). Those who failed were referred to the outpatient clinic for diagnostic BAEP before 1 month of age. This diagnostic evaluation also included tympanometry and ipsilateral AR research, behavioral observation audiometry, and otorhinolaryngological evaluation.	Audiologists, and technicians.	96.30%	N/A	4%	0.14%	2.99%
Weirather et al. 33	1 ^st^ and 2 ^nd^ phase: TOAE	NBs were tested with TOAE before hospital discharge. Those who did not pass the initial screening (stage 1) were brought for a new screening (stage 2) at 1 to 3 weeks of age. Babies who failed the phase 2 screening were referred to the hospital audiology department for a full diagnostic evaluation.	Neonatal unit staff member, audiologist (supervisor), program coordinator, and nursery “manager”.	99.70%	11%	N/A	N/A	N/A
Barsky-Firkser et al. 34	1 ^st^ phase: BAEP	Infants in regular nurseries were tested about 4 hours after birth. If the infant passed the BE screening, they were discharged with no further follow-up. If 1 ear failed, parents and pediatricians were instructed to reevaluate the infant at 6 months. If BE failure occurred, retesting was recommended at 3 months. With the NB who had risk factors for HL and those who were admitted to the Neonatal ICU, screening occurred shortly before discharge. Even if they passed the exam, they were referred for monitoring. The NB reassessment protocol consisted of: collection of the patient's clinical history, otoscopy, tympanometry, VRA, diagnostic BAEP, and OAE. If the patient had indications of conductive pathology, they were referred to an otolaryngologist and the exam was only completed after treatment.	Audiologist, and nurse	97%	9.6%	N/A	0.33%	N/A
Watkin 35	1 ^st^ and 2 ^nd^ phase: TOAE 2 ^nd^ phase: BAEP	The initial test was performed, whenever possible, as close to the time of discharge as possible. Babies admitted to the special care unit were tested as soon as they could leave the unit. Those who did not pass the initial BE test were referred to the hospital audiology department for retesting. Those who did not pass in 1 ear had the option to return for retesting. The initial test and retest were done by TOAE recording, and retest failures were referred for BAEP. If there were risk factors for deafness, parental concern, or delay in entering screening, failure of the initial TOAE test was followed by a BAEP without a retest of the TOAE. All infants referred for BAEP were followed up by the audiology service.	Technical assistants, audiologists, and administrative assistants.	80.80%	13%	1.75%	0.19%	N/A
Maxon et al. 36	1 ^st^ and 2 ^nd^ phase: TOAE 3 ^rd^ phase: BAEP	Initial TOAE screening obtained shortly before discharge, with most infants from regular nurseries tested between 24 and 72 hours of life. Babies discharged before 24 hours after birth were also screened shortly before leaving the hospital. Infants in the Neonatal ICU were tested when deemed medically stable. Infants who passed the screening in BE were disconnected from the program. Those who failed the TOAE test returned to the screening site in 4 to 6 weeks for retesting. If the retest was unsuccessful, they were screened with BAEP. Those who failed the BAEP at 30 dBHL (but passed at higher intensity levels) were referred for a behavioral diagnostic audiological evaluation, performed 6 months after the initial screening. Infants who failed the BAEP at intensities > 60 dBnHL were referred for a diagnostic BAEP, performed within 1 month of the Stage 3 screening.	Technicians (screening) and audiologist (diagnostic evaluation).	4,253 screened	7%	1.06%	N/A	N/A
Abdul et al. 37	1 ^st^ and 2 ^nd^ phase: DPOAE 3 ^rd^ and 4 ^th^ phase: BAEP-A	- Babies without risk factors for hearing loss: Hearing was examined before hospital discharge (usually 24 hours after birth). If the baby passed the 1 ^st^ stage of the examination with DPOAE, they were referred to the 2 ^nd^ stage, performed 2 to 3 months later, again with DPOAE. If the baby passed, they were referred to the 3 ^rd^ stage of screening, performed at school entry, at age 6. If the NB failed the 1 ^st^ test, they were referred for another test with DPOAE in the following 2 weeks, at the screening unit. Passing the retest placed the NB in the screening flow mentioned above. If the result was still a “failure”, the baby should undergo another screening, using BAEP-A. Approval in this phase would place the NB in the process. If, after the BAEP-A, the result persisted, the infant was submitted to another test with BAEP-A. Passing results placed the infant in the above-mentioned protocol; however, if they failed the BAEP-A again, he was referred to a speech therapist for a diagnostic evaluation in 2 to 3 weeks. - Babies admitted to the Neonatal ICU or with RFHL: Babies who stayed < 48 hours in the Neonatal ICU started screening in the 2nd stage of DPOAE testing, and if the results were normal, they followed the main flow. If the baby remained > 48 hours in one of the units, DPOAE and BAEP-A should be performed, according to the 1 ^st^ stage. In case of failure, another test with DPOAE and BAEP-A should be performed in the 2 ^nd^ stage (within 3 months). A 3 ^rd^ stage should be conducted with DPOAE and BAEP-A around 1 year, and a 4 ^th^ stage should be done at school age, using diagnostic-BAEP and complementing it with the opinion of a speech therapist.	Screening technicians, senior technicians, AVT and speech therapist, physicians, and administration team.	96.38%	30.80%	N/A	1.84%	N/A
Khandekar et al. 38	1 ^st^ and 2 ^nd^ phase: TOAE 3 ^rd^ phase: BAEP	Screening took place 24 to 48 hours after birth. Those who failed were tested again before leaving the maternity. If there was suspicion of HL, the NB was referred to an otolaryngologist at the same hospital. The otolaryngologists performed meatoscopy and TM status review. The neonates that were not screened in the maternity hospitals were tested at the time of vaccination visits. In case of failure, ENTs repeated the test after 6 weeks. Those who failed the retest were referred to the rehabilitation unit, where they were retested with TOAE and BAEP. If they failed, they were referred to a tertiary center for further investigations.	Nurses.	2002: 25.68% 2003: 53.41%	2002: 11% 2003: 10.70%	2002: 0.24% 2003: 0.26%	2002: N/A 2003: 0.12%	2002: 83.70% 2003: 89%
González De Aledo Linos et al. 39	Babies without RFHL: Phase 1 and 2: OAE 3 ^rd^ phase: BAEP Infants with RFHL: 1 ^st^ phase: BAEP	- For neonates without RFHL: 1 ^st^ phase (“screening”): OAE; 2 ^nd^ phase (“confirmation”): OAE; 3 ^rd^ phase (“diagnosis”): BAEP; 4 ^th^ phase (“treatment”); 5 ^th^ phase (“evaluation”). - In infants with RFHL, BAEP was performed from stage 1.	Hospital staff (not specified), nurse and auxiliary.	98.40% (1st phase) and 99.50% (2nd phase)	6,70%	0.7%	24.80%	2.50%

Abbreviations: AEP, Auditory Evoked Potentials; AR, acoustic reflexes; BAEP, Brainstem Auditory Evoked Potentials; BE, both ears; DPOAE, Distortion Product Otoacoustic Emissions; ENT, otorhinolaryngology; HL, hearing loss; ICU, Intensive Care Unit; ICU, Intensive Care Unit; NB, newborn; OAE, Otoacoustic Emissions; RFHL, Risk Factors for Hearing Loss; SPU, São Paulo Unit; TM, tympanic membrane; TOAE, Transient Otoacoustic Emissions; VRA, Visual Reinforcement Audiometry.

### Economic Analysis

All the included studies mentioned a cost-effectiveness analysis, but none presented statistical elements for the analyzes described or to support the economic analyzes based on indicators better known in Health Economics, which are part of the Health Adjusted Life Years (HALY) group. The objective of this group of indicators is to present the well-being of the individual in the form of years, also considering the quality of life experienced. Included within HALY are Quality-Adjusted Life Years (QALY), an indicator that represents the well-being of the individual, and Disability-Adjusted Life Years (DALY), which calculates not the social utility of the individual, but rather their disability. Both are widely used as a cutoff point to identify acceptable strategies in the cost-effectiveness ratio. On the other hand, the ICER is an important incremental data, since it compares two different scenarios and guides resource allocation considering the best one. But this metric was not reported in the reviewed studies either.

The costs presented were reported in different local currencies, requiring first conversion and then deflation to allow some sort of comparison. The amounts were reported in international 2012 dollars.

The most present information in the reviewed studies were the following: expenses of the NHS program, expenses with the first hearing assessment of NHS, with retesting, diagnosis, intervention, and operational expenses – those related to the expected expenses to produce products and services. The sources of funding for identified UNHS programs, usually from national or local research or projects. Specific information on economic analysis identified in the implementation of NHS programs is in Table 3 .

**Table 3:** Presentation of the economic analyzes performed in each article reviewed

Author	Expenditure of the NHS program	Screening fee (individual $)	Total cost in equipment	Initial screening expense	Retest Expense	Diagnostic expenditure	Intervention expenditure	Operating expenditure 1	Team time/salary	Administrative expenditure 2	Financing
Chen et al. 17	N/A	N/A	N/A	U$ 10.95 million	U$ 846.60 thousand	U$ 1.09 million	ISADs: U$ 19.45 million CI: 10.64 million	U$ 32.39 million	N/A	N/A	National Natural Science Foundation of China and National Pillar Program of Science and Technology
Wong et al. 18	U$ 9.342 - U$ 22.350	N/A	N/A	U$ 4.500	N/A	U$ 203.00 (single)	Annual rehabilitation costs: $1,846 (individual)	ISAD and batteries: U$ 380.00 - U$ 523.00 (individual) Equipment maintenance: $2,000	U$ 192,00 - 13.200.	N/A	Committee for the Protection of Human Beings Institutional Dartmouth College Review Board
Gupta et al. 19	3.79.127 INR (annual)	231 INR	1.25.357 INR	N/A	N/A	N/A.	N/A	Electricity: 4,994 INR Consumables: 601 INR	2.40.000 INR	N/A	Indian Council of Medical Research
Bevilacqua et al. 20	U$ 26,940.47 (annual)	U$ 7,00	Audix: $958.96. Software: $582.54. $660 Graphics: $582.54. I don't know if we leave all the equipment here: U$9.587,00.	N/A	N/A	U$6.850,47	N/A	U$ 1,278.30 (annual)	Speech Therapists specialized in Audiology: U$9,418.87 Non-specialized in Audiology: U$7,190.94.	N/A	Unified Health System
Mezzano et al. 21	NB without RFHL (total): 527,949 euros (OAE + BAEP) NB with RFHL (total): 192,144 euros (BAEP) Total: 720,093 euros	OAE (without RFHL): EUR 13.32 OAE + BAEP (without RFHL): EUR 16.58 BAEP (with risk): EUR 415.90	OAE: 78,000 Euro (total); 26,000 Euro (annual)	In 3 years: 404,271 euros (OAE) 34,755 (OAE) (annual) 415.90 euros (BAEP - RFHL) (annual)	EUR 41,226 (annual)	Population without RFHL: €32,951 (per identified case) Population with RFHL: €11,303 (per identified case).	N/A	Consumables and concerts: 19,492 euros (total); 6,497 euros (annual)	228,931 (total); 76,310 (annual). For retest: 2,048 euros (total); 682 euros (annual)	N/A	Italian Association for Research on Disabilities and Italian Ministry of Health
Olusanya *et al.* 22	Hospital program: $17,695 Community program: $15,262	Hospital Program: US$ 13.30 Community program: $7.62	Echo-Screen TS: $3,750 (unit). ALGO Portable: $15,625 (unit). Computer, printer, accessories, and software: $2,000. Total cost (3 Echo-Screen, 2 ALGO Portable, and 1 computer): $44,500.	N/A	N/A	Both programs: US$231.00 (per child)	N/A	Echo-Screen Olives: $133 (hospital) and $200 (community). Disposable electrodes for BAEP: US$ 4,824 (hospital) and US$ 1,668 (community).	Team of 2 programs: US$18,230. Project Coordinator: $9,000. Nurse's aide: $3,000 Support staff: $2,400 Support staff member: $2,400 Data entry clerk: US$1,500	Printing of materials (leaflet and posters informing about the screening program and consent form: US$ 0.15 per baby	Local charity organization (Hearing International Nigeria [HING]).
Ciorba et al. 23	Using ILO 92: U$ 17,800 (annual) Using AOAE: $17,966.67 (annual)	Using ILO 92: U$ 14.12 Using AOAE: U$ 13.86	Using ILO 92 Equipment: U$ 10.000 (unit) Computer and software: $4,000 (unit) Using AOAE Equipment: $5,000 (unit) Computer and software: $4,000 (unit)	N/A	N/A	Using ILO 92: U$ 3,137.14. Using AOAE: U$ 3.080.	N/A	Using ILO 92 Disposables: U$2000 (yearly) Using AOAE Disposables: $2,000 (annual)	Using ILO 92 Wages: US$12,000 (annual) Using AOAE Wages: $12,500 (annual)	Using ILO 92 Administration: $1,000 (annually) Using AOAE Administration: $1,000 (annual)	Regional Project by UNHS and the company LABAT Srl.
Cao-Nguyen et al. 24	US$ 71,500 (annual)	US$ 21	EchoScreen, AccuScreen: CHF 6,250 (unit) Biologice notebook: CHF 31,500 (unit) Computer program: CHF 10,000 (unit)	N/A	N/A	N/A	N/A	Calibration/disposables: CHF 300 (annually)	Nurses and ENT specialists: CHF 76.00 (annually)	N/A	Geneva University Hospital.
Lin et al. 25	Initial stage: $215,046 Final stage: U$ 189,193	Initial stage: U$ 10.10 Final stage: $8.90	TOAE: U$ 16.130 BAEP: U$ 29,030	Initial stage: U$ 6.37. Final stage: U$ 7.73.	N/A	Early Stage: $79,561 (total); $3.74 (per baby). Final Stage: $24,677 (total); $1.16 (per baby). Each test with diagnostic BAEP: U$ 64.50	N/A	N/A	For both stages: $119,355	N/A	Mackay Memorial Hospital and Children's Hearing Foundation.
Connolly et al. 26	N/A	N/A	Equipment for NHS: U$17,500 Computer (data management): $1,500 Printer (data management): $300	N/A	N/A	U$5,074 per diagnosis. ($29,369 per diagnosis in the non-RFHL group and $1,284 in those with RFHL)	N/A	Electrodes: $9.75 Calibration/warranty: $0.10	Time: 1,880 hours/week. Wages: coordinator ($32.50), analyst ($17.88), clerk ($13.00), audiologist ($40.63)	N/A	N/A
Messner et al. 27	U$ 137.051 (annual)	U$ 27,41	ALGO II: $17,000 (unit); $10,200 (annual total).	N/A	N/A	N/A	N/A	Disposables: $8.65 (unit); $49,738 (annual total)	Wages: audiologist: $66,538 (annual)	Printing of reference cards for parents, educational materials, office supplies, sending reports to primary care physicians: $10,000 (annual)	N/A
Gorga et al. 28	U$ 58,028 (annual total)	U$ 26,38	U$ 23.500 (total)	$1.00 (per baby); $2,200 (annually)	$200 (per baby); $8,800 (annual total)	N/A	N/A	Disposables: $3,828 (annual)	Salaries and Benefits: $30 (per hour); $21,900 (annual total)	N/A	N/A
Isaacson 29	U$ 166.000	U$ 71.00	U$ 6.000 (total)	N/A	N/A	U$ 23.500 (total)	N/A	Disposables, educational and marketing materials: $5,000 (total)	U$ 130.000 (total)	N/A	National Organization for Hearing Research, The Ronald McDonald House Charities and a private family donation
Kanne et al. 30	U$ 56.045	U$ 24.48	Equipment, supplies: U$ 20.000	N/A	N/A	$11,209 (per baby)	N/A	N/A	Time: audiologist: 17 hours/week; Technical team: 10 hours/week Salary: audiologist: $21,760; technical team: $6,400 fringe benefits (28% of salary): $7,885	N/A	N/A
Bantock et al. 31	GBP 61,400	N/A	ILO88 Equipment: £6,000 (unit); £36,000 (5 units).	N/A	N/A	GBP 355 (per test); GBP 4 500 (annual total)	N/A	Probes: £130 (unit); £3,900 (annual total) Olives: GBP 1 000 (total)	Nurses: £39,000; administrative assistants: £7,080; annual total: £46,000.	N/A	N/A
Mason *et al.* 32	1 ^st^ phase: U$ 179.000 (total 5 years) 1 ^st^ and 2 ^nd^ phases: U$ 266.300 (total 5 years)	1st phase: U$ 17,00 1st and 2nd phases: U$ 17.750 Per loss? Or how much does it cost to find that loss?	Reusable equipment (BAEP-A): U$ 11.000 (total 5 years) Reusable equipment (BAEP-Diagnostic): U$ 5.000 (total 5 years)	U$ 179,000 (total 5 years); U$ 17.00 (individual)	N/A	Behavioral Audiometry: U$ 6,400 (total 5 years); U$ 30.00 (individual) BAEP-Diagnostic: U$ 37,200 (total 5 years); U$ 120.00 (individual) Tympanometry: U$ 4,400 (total 5 years); U$ 15.00 (individual)	N/A	Disposables: U$ 59.000 (total 5 years)	Salaries (total 5 years): technician: $109,000; audiologist/administrator: $26,000.	N/A	Health Insurance. Non-members pay U$ 30.00.
Weirather et al. 33	U$ 2.858,62	U$ 7.42	U$ 446.00 (total)	N/A	N/A	N/A	N/A	Disposables: cost of replacing probe every 750 babies, computer supplies and paper): $416.97 (total)	Time: 118.07 hours. Wages: $7.00 - $16.00 (per hour); $1,168.63 (total). Additional Benefits (30% salary): $350.59. Overload (20% costs): $476.44.	N/A	N/A
Barsky-Firkser et al. 34	U$ 149.760	U$ 29,95	Nicolet compass: $6,400	N/A	N/A	N/A	N/A	Disposables: U$ 25.000	Full-time audiologists: $100,000 Daily rate for 1 audiologist: U$ 6.500. Secretary (15 h/week): U$ 9.360	N/A	N/A
Watkin 35	44,218 pounds (annual).	9.80 pounds	ILO88: £4,560 (annual) BAEP: £1,900 (annually) computer + software: £220 (annually)	N/A	N/A	4,900 pounds (each case)	N/A	Stationery, probes, electrodes: £3,100 (annual)	Audiologist: £6,200 (annual), technical assistant: £8,424 (annual); senior technical assistant: £14,179 (annual); administrative assistant: £5,635 (annual).	N/A	N/A
Maxon et al. 36	U$110.775	U$26.05	3 TOAE devices 1 for BAEP 4 computers 2 printers Amortization in 5 years: U$ 6,575.	N/A	N/A	U$4,378 (each case)	N/A	Disposables: $12,006	Average time worked: Screening (103 h/s); clerical (60 h/s); audiologist (60 h/s); coordinator (20 h/s). Salaries (total): $60,654. fringe benefits (28% of salaries): $16,983. Overload (29% of salaries): US$14.557	N/A	Maternal and Child Health Program, Health Resources and Services Administration, Department of Health and Human Services.
Abdul et al. 37	N/A	N/A	DPOAE (Audex): 30,000 QR (unit); 300,000 QR (total, 10 units). DPOAE (Echoscreen): 30,000 QR (unit); 120,000 QR (total, 4 units). BAEP-A (GSI): 60,000 QR (unit); 120,000 QR (total, 2 units). BAEP-A (Abeer): 90,000 QR (unit); 180,000 QR (total, 2 units). Diagnostic-BERA: 100,000 QR (total, 1 unit). Diagnostic BAEP (Vivasonic): 66,000 QR (unit); 198,000 QR (total, 3 units).	N/A	N/A	20,218.98 QR (each case)	N/A	Disposable electrodes: 24,000 QR (annual); 144,000 QR (total).	Triage technicians (12): 3,800 QR/month and 45,600 QR/year; technician (6): 8,000 QR/month and 48,000/year; senior technicians (5): 6,000 QR/month and 30.000 QR/year; AVT and speech pathologist (5): 9,600 QR/month and 48,000 QR/year; physicians (5): 50,000 QR/month and 250,000 QR/year; administration staff (4): 3,400 QR/month and 136,000 QR/year. TOTAL (6 years): QR 3,355,600.	N/A	N/A
Khandekar et al. 38	U$ 155.475	U$ 7.10	Per unit: $4,250; total: $114,750 (27 units)	N/A	N/A	N/A	N/A	Disposable olives: U$ 150 (600 pack); U$ 4.500 (total, of 22.000 olives).	Phase 1: $750/month Phase 2: $1,500/month; Phase 3: $1,500/month; Total: U$ 36.225	N/A	N/A
González De Aledo Linos et al. 39	N/A	EUR 1.30	N/A	N/A	N/A	EUR 867	N/A	N/A	N/A	N/A	N/A

The original values have been retained for reference purposes.

All values that did not present costs in dollars were converted using the PPP rate. For inflation adjustment, the Gross Domestic Product index of 2012 was applied, given that the last article selected dates back to this year's economic analysis, minimizing possible distortions in the evaluation.

1 Operating expenditure: expenditure to produce products and services.

2 Administrative expenses: general expenses that are not directly related to production.

CHF: Swiss Franc; €: euros; INR: Indian Rupee; Int$: International $; £:Pound sterling; QR: Qatari Real; R$: Brazilian Real; US$: United States Dollar.

### Overview of The Studies

There was no divergence between the reviewed studies regarding the positive cost-effectiveness ratio, considering the implementation of UNHS programs. There is an assertion of the use of these data to improve the programs and identify their weaknesses. The importance of reflecting on the cost-effectiveness of UNHS is perceptible, given the scarcity of epidemiological data on issues of this practice, such as the prevalence of hearing impairment in the population, and what is attributable to congenital hearing loss, often not detected and not treated early. And also due to the scarcity of audiological care data in children with Risk Factors for Hearing Loss (RFHL), among others.

Having a data system that associates the initial results of UNHS and the diagnostic assessments made in rehabilitation services is still a challenge for the best settings in the implementation of UNHS. Few articles mention information on data storage of UNHS programs and those that exposed this information were usually linked to databases of the very places of execution of this screening. In these sites, plausible factors for improvement were identified, such as control of neonate follow-up, logistical issues, and the false-positive rate. Table 4 identifies the conclusions of the economic analysis of each study reviewed, looking for positive and negative points and perspectives, as well as how UNHS data is stored.

**Table 4:** information on data storage of NHS and conclusions of the economic analysis of each study reviewed

Author	Form in which the NHS data are stored	Pros	Cons	Future
Chen et al. 17	System integrated with the Shanghai Municipal Health and Family Planning Commission.	Early intervention is a comprehensive and key measure to help hearing-impaired children regain their hearing. Economic evaluation of UNHS, such as cost-benefit analysis, plays an important role in aiding policy making and is justifiable in Shanghai.	The administrative costs of the program were not measured separately at the time of the calculations, and the labor costs of program administrators were included.	There is still room for improvement about educational rehabilitation and the creation of a better infrastructure system
Wong et al. 18	N/A.	The cost-effectiveness of OAE-based infant hearing screening in Jinotega has been proven.	Relatively small sample size, which may limit the potential to demonstrate statistical significance. In addition, only half of the infants who failed screening took the retest, so one can only estimate the true prevalence of HL.	N/A.
Gupta et al. 19	Electronic database in MS Access.	Finding that UNHS is feasible for implementation.	High rate of “lost” NBs during follow-up, less than 95% coverage and high rate of retest referrals. Significant loss of babies during follow-up and lack of linkage to rehabilitation services is likely to limit its usefulness in any setting, and until such time as these factors are addressed, UNHS may not be cost-effective compared to screening based on the presence of RFHL.	It is important to reflect on the cost-effectiveness of UNHS compared with restricted screening. Having a dedicated team to perform UNHS is necessary for the establishment of any universal screening program in any facility.
Bevilacqua et al. 20	N/A.	The NHS performed by SUS is feasible and allows reaching reference values and quality indicators established by the *Joint Committee on Infant Hearing* . In the sample, HL was identified very early, regardless of degree, and treatment with hearing aid and/or CI was offered to all diagnosed children.	High rate of non-follow-up in the program.	A more efficient guidance to families and greater control of hospital discharge are needed to increase the number of NB included in NHS. Further steps for neonatal hearing health actions should be developed to identify HL in infants whose families dropped out of the program, false-negative rates, and acquired HL.
Mezzano et al. 21	N/A.	The cost-effectiveness of the program proved to be favorable.	N/A.	To understand the real long-term financial effects of UNHS, more evidence is needed on the impact of early intervention on language and on subsequent changes in educational cost and lifetime social costs.
Olusanya *et al.* 22	N/A.	A two-stage UNHS program with TOAE/BAEP is feasible in hospital and non-hospital settings in a developing country.	High rate of NB “lost” during follow-up. In addition, costs associated with the support of children detected with hearing loss, such as the provision of hearing aids, offered free of charge by these screening programs, were not considered.	Applying measures to minimize NB loss during the process, including culturally appropriate public education and reduction in the number of visits from screening to diagnosis, should lead to improved program cost outcomes.
Ciorba et al. 23	Own software.	The information presented in the article should simplify decision making by health policy makers regarding mandatory state NHS strategies.	One of the most important issues to consider in a universal screening program is reducing the “false positive” rate, which can create discomfort.	Program coverage may increase significantly in the future using a database to track/follow up cases referred to outpatient clinics.
Cao-Nguyen et al. 24	N/A.	The elapsed time for diagnosis and rehabilitation has been reduced since the implementation of the UNHS program at the institution.	N/A.	N/A.
Lin et al. 25	N/A.	The use of the BAEP-A after TOAE reduced the number of patients sent for additional diagnostic testing, thereby decreasing the overall program expense.	High rate of NBs without follow-up during follow-up.	N/A.
Connolly et al. 26	Database of the neonate nursery and Neonatal ICU, database of the Early Auditory Selection and Intervention Service and individual medical records.	The adopted protocol was successful in screening program of virtually all neonates, providing BP diagnoses and timely interventions.	There are still problems in obtaining early diagnosis and intervention, with loss to follow-up.	Future research will compare the proportion of high-risk patients to those at low risk statewide. Prospective studies will be developed to evaluate the cost effectiveness of the current protocol compared to dual instrument screening (OAE + BAEP).
Messner et al. 27	Hospital Medical Record System.	Great availability on the part of the volunteers, so that there is seldom any downtime of the activities.	There were no significant cost savings during the first two years of the program.	N/A
Gorga et al. 28	N/A.	The protocol in which all infants are screened with DPOAE, followed by BAEP testing only for those who fail, is an efficient and cost-effective means of screening for HL.	The higher failure rates may be a consequence of OE and ME status early in life and as a function of when screening was conducted. It is also difficult to see how cost estimates for UNHS developed in one state or hospital can be applied to other states or hospitals where the protocol, number of infants and targets may be different.	Administrative overheads have not been considered in the current cost estimates but should be considered if the intention is to provide a cost-effective service.
Isaacson 29	Database based on Microsoft Access.	With adequate resources, UNHS can be conducted in urban settings and with distinctive settings where many of the North Americans live.	N/A.	Future goals are to reduce the false-positive rate, speed up access to amplification/CI and reduce the cost of testing.
Kanne et al. 30	N/A.	The use of TOAE in two-phase NHS suggests that this method is cost-effective and, when administered by a trained team, reproducible, objective, easy to perform, minimally invasive, and faster than BAEP.	Not being able to screen all babies born in the hospital and the difficulty of accessing all babies in the Neonatal ICU, due to inconsistent references of the team. Lack of TOAE effectiveness when compared to targeted screening. Lack of funding and difficulties with program implementation.	N/A
Bantock et al. 31	Regional Interactive Child Health System.	N/A	Scarce coverage.	Compare community NHS with the cost of neonatal screening at the three hospitals in the district, as well as a nearby facility where a small but significant number of children are born.
Mason *et al.* 32	N/A.	The cost-effectiveness of the UNHS programme is favourable.	N/A.	It is believed that more research is needed for infants with transient and fluctuating HL, as this group may benefit from temporary amplification.
Weirather et al. 33	HI*SCREEN 96 software.	The use of HI*SCREEN 96 software has not only increased accuracy but has substantially reduced the staff time associated with these tasks. Having a screening program operating directly in the nursery is much more efficient than having a separate room where babies are brought for screening.	N/A.	N/A.
Barsky-Firkser et al. 34	N/A.	The data demonstrate the feasibility, efficacy, benefits, and necessity of using conventional BAEP screening for a one-stage procedure. One-stage screening with BAEP is believed to save time and money.	N/A.	N/A.
Watkin 35	Internal database.	Cost-effectiveness of feasible UNHS.	Methods to identify progressive and acquired hearing losses later in childhood are still considered necessary.	UNHS programs using TOAE should increase specificity without losing sensitivity.
Maxon et al. 36	N/A.	UNHS is not only feasible, but the relatively low costs make it very practical.	N/A	Advancing the implementation of UNHS.
Abdul et al. 37	N/A.	Compared to similar international UNHS programs, it is being effective.	False-positive results produced inevitable concern among parents.	Reduce HL rates by emerging work in genetics and chromosome studies of the local population in Qatar. Improve the sensitivity and specificity of the equipment in use.
Khandekar et al. 38	Info-bank-ORACLE” software.	Universal hearing screening in Oman could detect HL at a very early age.	Variations in coverage between regions of the country, high false-positive rates, and logistical problems.	Reduce program costs by reusing olives after sterilization; reduce loss of NBs to follow-up by providing more guidance to parents and delivering informational materials. Possibility of higher yield when hearing screening coverage improves.
González De Aledo Linos et al. 39	N/A.	Key figures for phases 1 and 2 are of good quality, with a good coverage rate and low levels of referrals for retesting.	Evasion was higher among infants with RFHL than those without risk (may be due to multiple comorbidities of these babies or undocumented deaths).	There are still difficulties in establishing, in practice, the appropriate start of treatment.

Abbreviations: BAEP, Brainstem Auditory Evoked Potential; BAEP-A, Automatic Brainstem Auditory Evoked Potential; CI, cochlear implant; DPOAE, Otoacoustic Emissions: Distortion Product; HL, hearing loss; ICU, Intensive Care Unit; ME, middle ear; NB, newborn; NHS, Newborn Hearing Screening; OAE, Otoacoustic Emissions; OE, outer ear; RFHL, Risk Factors for Hearing Loss; SUS, Unified Health System; TOAE, Transient Otoacoustic Emissions; UNHS, Universal Newborn Hearing Screening.

## Discussion

According to the Joint Committee on Infant Hearing (JCIH), BAEP and OAE tests are recommended for screening children's hearing. The OAE is indicated for early identification of hearing impairment in newborns, and the BAEP-A should be done when, regardless of the results of the OAE test, the newborn has any RFHL, or for newborns who have failed the OAE. But there are several choices in the organizations of UNHS programs and in the design of protocols, as reported in the included studies. In a recent study 40 it was possible to identify that a two-stage OAE-BAEP-A protocol results in referrals for diagnostic tests four times more frequently when compared with the three-stage OAE-OAE-BAEP-A protocol. On the other hand, the application of several screening stages may increase the number of lost cases of hearing impairment in each assessment stage, either by non-attendance of the babies in the next assessment or by false-negative results.

This diversity may stem from the various existing programs, which may make it difficult for countries and institutions wishing to develop UNHS programs according to a single protocol to join, as they generally seek examples to serve as a guide for implementation. These differences in protocols should not confuse but rather guide the use and development of relevant protocols, ensuring that implemented UNHS programs reach benchmark standards. There are many reasons why countries choose one recommended protocol and not another. These relate to social context, available resources and constraints imposed in health settings. But the final choice should be limited not by resource constraints, but by the current evidence in the literature.

In addition to the type of program implemented, it is important to analyze its coverage. This is a qualitative measure, indicating that UNHS is available to all eligible infants born at the administering hospital or living in the administering region or country. Therefore, this reflects the quality of access and adherence to the UNHS program. There is inherent variability in the quality and validity of data in the selected studies, as program coverage encompassed different percentages. Of the 23 studies selected, 18 reported the percentage of coverage. Among these, three showed coverage ≥ 95% in their entirety and 1 only in the first phase. Five studies presented indexes ≥ 97%, and only 1 reported this index in the second stage. In the other articles, the index ranged between 25.68 and 93.60%.

It is extremely important that UNHS program managers control quality indicators through the percentage of all newborns who complete this assessment within the 1 ^st^ month of life, as well as all newborns who did not pass the initial hospital screening and required a new outpatient assessment, those who did not pass any subsequent new checks before audiological assessment, and the percentage of newborns who did not pass the initial screening and were then reassessed, 5 among others. Quality indicators for each phase are defined by scientific institutions and should be used as a parameter to help control the effectiveness of programs, determining whether interventions represent an improvement, and to ensure that improvements are sustained.

Methods for identifying areas for improvement, evaluating the selected protocol design, and ensuring the effective use of resources should be decisive components in all UNHS programs. The quality of the analysis ensures the support needed to achieve public policy efficiency and is directly related to the quality of the management system for the data explored in the research. A performance evaluation program that intends to present complete results, with a deep difference in costs and patient monitoring, requires an efficient organization of information. The absence of such a system may impair data accounting and compromise the evaluation of the flow, a factor that was evident in the heterogeneity and the omission of data found in some selected articles. It is not enough just to implement the program, but also to evaluate its performance in relation to costs, benefits to users and savings in the use of health resources, for example.

For countries implementing UNHS, the only method of ensuring effective use of resources is to collect data, monitor, and evaluate screening program performance, which can be done through digital platforms with broad access and communication. Thus, the quality of the program data management system is very important, and this aspect is still a cause for concern, as many structured programs do not monitor or evaluate their performance.

From the present review, it is clear that, in different countries, the professionals responsible for UNHS are also distinct, which includes trained volunteers or certified professional screeners. Detailed requirements for the training and skills development of these assessors are provided by some adopted protocols. The JCIH guidelines present comprehensive guidance on the role and competencies of different professionals as part of a multidisciplinary team approach, including audiologists, physicians, and nursing staff, to ensure the delivery of UNHS. This emphasizes the importance of the audiologist experienced in the assessment of newborns and young children, and the supervision of each component of the hearing screening program, especially in its implementation and, whenever possible, in each hospital.

In the present study, no statistical economic analysis was found that could prove the evidence of the cost-effectiveness of the programs. Only self-reported information on this cost-effectiveness relationship was found. Therefore, it is important to highlight that the principle of optimization and investment begins with economic analysis, and the impact on health can be considered when a budget is analyzed, or the minimization of costs considering an outcome. The present study found that the implementation of UNHS programs can have a positive impact on health services by identifying hearing loss early. Thus, they are cost-effective, especially for their results that have repercussions throughout the lives of the individuals. It is important to stress the importance of defining the context and its characterization, as well as the selection of the protocol to be used for structuring the UNHS program. On the other hand, as more countries and organizations develop their UNHS programs, there is a constant review of existing information and benefits of its applicability. 41

## Conclusion

In analyzing the data, the expectation was to find the following: the expected mean QALYs and costs for the base-case cohort, the ICER to analyze screening strategies, and information on DALYs, with data on disability-adjusted years of life lost, to ensure that the intervention is cost-effective.

Hence, one can see the importance of including this unaddressed information, as these data show where governments need to act to strengthen their health systems and protect people from health care consequences and potential costs.

Even with the constant omissions of data, the results of the present review indicate that UNHS programs are generally cost-effective; that is, they state that the orientation to implement UNHS programs by analyzing resource allocation and comparing the efficiency of proposed interventions is positive.
