# Supplementary material for: Cost-effectiveness of Neonatal Hearing Screening Programs: Systematic Review
Source: Int Arch Otorhinolaryngol. 2024 Apr 9;28(4):e668–96. doi: 10.1055/s-0043-1776703 (PMC11511281; doi:10.1055/s-0043-1776703)
Supplement: Supplementary file 1 — Supplementary Material [file 10-1055-s-0043-1776703-s2023011469sr.pdf]

Appendix 1 - Literature Search Protocol

Cost-benefit analysis of Universal Neonatal Hearing Screening Program: systematic review

The objective of this scoping review is to determine how cost-effective Universal Neonatal Hearing Screening Programs are.

PICO

P – Newborns

I – Universal Neonatal Hearing Screening Program

C – No Universal Neonatal Hearing Screening Program

O – Evidence of cost-effectiveness

Inclusion criteria

Studies that include the following information:

1. Studies published so far, with no date and language restrictions;
2. Articles in which the analysis is exclusively from Universal Neonatal Hearing Screening;
3. Studies that contain, in their results, data of cost-benefit analysis of the insertion of the Universal Neonatal Hearing Screening Program.

Exclusion criteria

1. Duplicate or unavailable articles;
2. Articles in which the objective is to compare methodologies for the realization of Universal Neonatal Hearing Screening;
3. Articles in which only one section of the Universal Neonatal Hearing Screening Program is presented;
4. Articles that limit the population analyzed in carrying out Universal Neonatal Hearing Screening;
5. Studies that focus only on preschool or school hearing screening programs.

Sources

Open to any existing literature, including observational studies, as like case studies, serie of cases, case-control and randomized clinical trials. The studies that will be excluded are literature reviews, systematic reviews or meta-analysis.

Search strategy

The search strategy will be performed in 4 steps:

1. An initial search for articles related to the cost-effectiveness of Neonatal Hearing Screening. Keywords and index terms were identified in the articles, verified in the Medical Subject Headings (MeSH), and a strategy created for the second formal search in the databases.
2. The formal search was performed in the following databases: PubMed / MEDLINE, Scopus, Web of Science, LILACS, Embase, Cochrane and CINAHL. This search used the keywords and terms from the index, or the closest approximations according to the database.
3. The references of each article included in the formal search were reviewed and articles of interest were extracted for analysis.
4. Articles that met the inclusion criteria after reviewing the titles and abstracts were fully analyzed and included in the final review.

Articles were not limited by language, geographic region or publication date. This search algorithm was performed independently by two study authors, based on the exclusion and inclusion criteria. A third person was the judge, who was responsible for verifying and analyzing the results obtained by the two evaluators. If, in any way, the two reviewers disagree, the decision will be made between them and the judge. Each reviewer will record all extracted data in a spreadsheet that has been sent to the judge separately. This organized a new spreadsheet in order to assess the compatibility between the information.

Data reporting/Analysis

This review was formatted and organized according to the checklist of preferred reporting items for systematic reviews and meta-analyses for scope reviews (PRISMA-ScR). The objective is to characterize the current state of cost-effectiveness research in newborn hearing screening programs and to identify gaps in the literature.

Appendix 2 - Search Algorithms

PubMed

(Neonat* screening* OR Newborn* screening*)

AND

(Costs and Cost Analysis OR Cost Control* OR Economics OR Cost-Benefit Analys* OR Cost-Effectiveness Analysis)

(("neonat*"[All Fields] AND "screening*"[All Fields]) OR ("newborn*"[All Fields] AND "screening*"[All Fields])) AND "hear*"[All Fields] AND ("costs and cost analysis"[MeSH Terms] OR ("costs"[All Fields] AND "cost"[All Fields] AND "analysis"[All Fields]) OR "costs and cost analysis"[All Fields] OR (("economics"[MeSH Subheading] OR "economics"[All Fields] OR "cost"[All Fields] OR "costs and cost analysis"[MeSH Terms] OR ("costs"[All Fields] AND "cost"[All Fields] AND "analysis"[All Fields]) OR "costs and cost analysis"[All Fields]) AND "control*"[All Fields]) OR ("economical"[All Fields] OR "economics"[MeSH Terms] OR "economics"[All Fields] OR "economic"[All Fields] OR "economically"[All Fields] OR "economics"[MeSH Subheading] OR "economization"[All Fields] OR "economize"[All Fields] OR "economized"[All Fields] OR "economizes"[All Fields] OR "economizing"[All Fields]) OR (("cost benefit analysis"[MeSH Terms] OR ("cost benefit"[All Fields] AND "analysis"[All Fields]) OR "cost benefit analysis"[All Fields] OR ("cost"[All Fields] AND "benefit"[All Fields]) OR "cost benefit"[All Fields]) AND "analys*"[All Fields]) OR ("cost benefit analysis"[MeSH Terms] OR ("cost benefit"[All Fields] AND "analysis"[All Fields]) OR "cost benefit analysis"[All Fields] OR ("cost"[All Fields] AND "effectiveness"[All Fields] AND "analysis"[All Fields]) OR "cost effectiveness analysis"[All Fields]))

Embase

(neonat* AND screening* OR newborn*) AND screening*

AND

(((costs AND ('cost analysis'/exp OR 'cost analysis' OR (('cost'/exp OR cost) AND ('analysis'/exp OR analysis))) OR

 © 2024. Fundação Otorrinolaringologia. All rights reserved.

'cost'/exp OR cost) AND control* OR 'economics'/exp OR economics OR 'cost benefit'/exp OR 'cost benefit') AND analys* OR 'cost-effectiveness analysis'/exp OR 'cost-effectiveness analysis' OR (('cost effectiveness'/exp OR 'cost effectiveness') AND ('analysis'/exp OR analysis)))

CINAHL

Neonat* screening* OR Newborn* screening*

AND

MH "Costs and Cost Analysis + " OR Cost Control* OR MH "Economics" OR Cost-Benefit Analys* OR MH "Cost-Effectiveness Analysis"

Cochrane

Neonat* screening* OR Newborn* screening*

AND

"Costs and Cost Analysis"[Mesh] OR Cost Control* OR "Economics"[Mesh] OR Cost-Benefit Analys* OR "Cost-Effectiveness Analysis"[Mesh]

Scopus

(neonat* AND screening* OR newborn* AND screening*)

AND

(costs AND cost AND analysis OR cost AND control* OR economics OR cost-benefit AND analys* OR cost-effectiveness AND analysis)

Web of Science

 © 2024. Fundação Otorrinolaringologia. All rights reserved.
